# EEG temporal–spatial transformer for person identification

**DOI:** 10.1038/s41598-022-18502-3

**Published:** 2022-08-23

**Authors:** Yang Du, Yongling Xu, Xiaoan Wang, Li Liu, Pengcheng Ma

**Affiliations:** 1 Big Data Center, Nanfang Hospital, Southern Medical University, Guangzhou, 510515 China; 2 Brainup Research Lab, Naolu Technology Co., Ltd., Beijing, 100124 China

**Keywords:** Computer science, Information technology

## Abstract

An increasing number of studies have been devoted to electroencephalogram (EEG) identity recognition since EEG signals are not easily stolen. Most of the existing studies on EEG person identification have only addressed brain signals in a single state, depending upon specific and repetitive sensory stimuli. However, in reality, human states are diverse and rapidly changing, which limits their practicality in realistic settings. Among many potential solutions, transformer is widely used and achieves an excellent performance in natural language processing, which demonstrates the outstanding ability of the attention mechanism to model temporal signals. In this paper, we propose a transformer-based approach for the EEG person identification task that extracts features in the temporal and spatial domains using a self-attention mechanism. We conduct an extensive study to evaluate the generalization ability of the proposed method among different states. Our method is compared with the most advanced EEG biometrics techniques and the results show that our method reaches state-of-the-art results. Notably, we do not need to extract any features manually.

## Introduction

In today’s globalized world of information, the security of personal information has become particularly important^1^, leading to the need for new and more sophisticated identification technologies. Even though existing identification technologies have widely applied in daily life and accomplished high accuracy, including fingerprints, iris, or face recognition^2–4^ and achieving high recognition accuracy rates. However, the problem with these biometrics is that they can be easily stolen or revealed inadvertently. The security of these technologies is not effectively guaranteed. Compared to conventional biometrics mentioned above, cognitive biometrics has attracted more research interest for its security reasons.

Unlike conventional biometrics, which relies on physiological or behavioral characteristics, cognitive biometrics is a type of biometrics that measures human brain activity and analyzes how people “think”^5^. There are various human brain activity measurement methods, and these methods are based on different principles to reflect brain activity. Functional magnetic resonance imaging (fMRI) measured the concentration of oxyhemoglobin and deoxyhemoglobin, which can indicate the hemodynamic changes caused by neuronal activity. Positron emission tomography (PET) measures neuronal metabolism by injecting a radioactive substance into the subject’s body. Near-infrared spectroscopy (NIRS) measures the concentration of oxyhemoglobin and deoxyhemoglobin by the intensity of reflection of infrared light from the cerebral cortex to reflect brain activity. Magnetoencephalography (MEG) collects the magnetic field generated by brain currents while electroencephalography (EEG) collects the electric fields generated.

We chose EEG for the identification task. Compared to other techniques, EEG can be acquired by portable and relatively inexpensive devices^6,7^. In particular, non-invasive brain–computer interface technology is often used to capture EEG signals, which is safer and more convenient than the invasive approaches. The amplitude of the EEG signal of normal humans ranges from 10 to 200 $\upmu$V, while frequency usually varies between 0.5 and 40 Hz. It has a high temporal resolution, usually in the order of milliseconds^5^. In terms of spatial resolution, EEG reveals a lower spatial resolution due to the size limitation of the acquisition device and the interaction of the electric fields among different brain regions. Yet it is worth noting that individual variability is the basis of person identification, and EEG is no exception. Some studies^8,9^ have demonstrated that EEG signals have strong individual variability, especially in alpha waves^10^. Consistency is another crucial factor for identification, as this biometrics requires test–retest, which means that the features stably remain invariant across time and place^11,12^. The EEG signal is also highly secure. This is especially important for person identification as person identification requires specialized acquisition equipment and amplifiers to collect information. Such personal information must not be inadvertently leaked or accessed remotely. Hence, data security-wise, EEG-based identification is reliable since it is more difficult for criminals to exploit. EEG ensures information security through emotion detection. Identification cannot be processed without users’ consent, as nervousness detected by EEG can lead to authentication failure. In addition, while the EEG signal is an internal trait that can only be generated when the brain is active, it naturally carries the function of liveness detection^13^. Last but not least, EEG signals are universal, and EEG signals can be captured from every individual unless some pathology causes structural damage to the brain that prevents the production of EEG signals.

In summary, EEG person identification shows great promise for application. However, most of the current research only studied the recognition in a single state, which is still unable to guarantee the accuracy and robustness of recognition. Therefore, we applied the attention mechanism to construct a network for identification tasks and made great progress. The main contributions of this paper are described below:

- We propose the transformer encoder-based neural network model ETST, EEG temporal–spatial transformer, which can commendably extract the information of EEG signals about individual differences in time and space domains well and ensure the accuracy of identification even in the case of cross-state.
- Extensive experiments are conducted and the results show that our model outperforms all state-of-the-art models. We investigate the role of temporal and spatial information of EEG signals on the person identification task. In addition, the effect of different position encoding on EEG transformer is investigated.
- We explore the effect of sample length on our transformer-based model and introduce a data augmentation method to improve the performance. The method increases the sample size by increasing the overlap rate between samples in time and an improvement of between 1 and 3% is observed with the strategy finally.

## Related works

The current *EEG-based biometrics systems* are broadly divided into two approaches. One is to extract distinguishable features first and then utilize traditional machine learning methods for classification, and the other is to employ an end-to-end deep learning approach, which accomplishes both feature extraction and classification. Kong et al. assume that task-related EEG can be decomposed into two parts, including background EEG (BEEG) and residue EEG (REEG). BEEG contains a person’s distinctive features whereas REEG is composed of task-evoked EEG and noises. Kong utilized the identification algorithm based on low-rank matrix decomposition (LRDM) to decompose the EEG signal and then used the maximum correntropy criterion (MCC) algorithm to accomplish the classification^14^. Wang et al. argued that the functional connectivity of the brain reflects individual specificity. They computed the connectivity of the EEG signal by calculating metrics of EEG signals as feature vectors and then used a discriminant model based on Mahalanobis distance to conduct person identification^15^. Moctezuma et al. adopted empirical mode decomposition (EMD) to decompose EEG signals into a set of intrinsic mode functions (IMFs), and subsequently selected the closest two IMFs and decomposed them into four features. In this way each channel will return eight features. Eventually, they employed support vector machine (SVM) with radial basis function (RBF) as a classifier^16^. Besides using SVM as a classifier, Alyasseri et al. applied FPA$\beta$-hc, which is a hybrid optimization technique based on binary flower pollination algorithm (FPA) and $\beta$-hill climbing to extract features^17^. Yıldırım et al. constructed a 1D CNN model stacked with multiple layers to extract deep-level features of EEG signals about individual specificity^18^. Wilaiprasitporn et al. tried to combine convolutional neural network (CNN) and recurrent neural network (RNN), where CNN is used to extract spatial features and RNN is used to extract temporal features^19^. Özdenizci et al. tried an adversarial inference approach within a deep convolutional network structure, which is able to learn session-invariant and person discriminative features^20^.

Currently, *Transformer* has shown good results in both natural language processing (NLP) and computer vision (CV) fields^21–23^. Transformer is able to model long-range dependencies and has a faster computation speed compared with RNN or long short-term memory (LSTM) because of its parallel computing characteristic. Therefore, Transformer has taken the lead in the NLP field, attracting interest from researchers. However, the ability of Transformer to process EEG signals has yet to be investigated by scholars. Arjun et al. directly migrated ViT, which performs well on images, to EEG signals. The EEG signal in 1D was cut into different patches in the time dimension and used as input to the ViT model^24^. Lee et al. combined EEGNet and transformer, using an EEGNet-based convolutional neural network to obtain the temporal–spectral-spatial features^25^. Tao et al. proposed a gated Transformer, which is a combination of the self-attentive mechanism and the gating mechanism in GRU to obtain the information of EEG signals on time series^26^. Song et al. proposed a method based on common spatial pattern (CSP) to extract the spatial features of the EEG signals along with a self-attention algorithm to decode them. This method achieves a state-of-the-art effect^27^. These approaches show that the self-attentive mechanism can improve the performance of brain–computer interface (BCI) systems. Therefore, we designed our model based on the self-attention mechanism.

## Methodology

**Figure 1:**
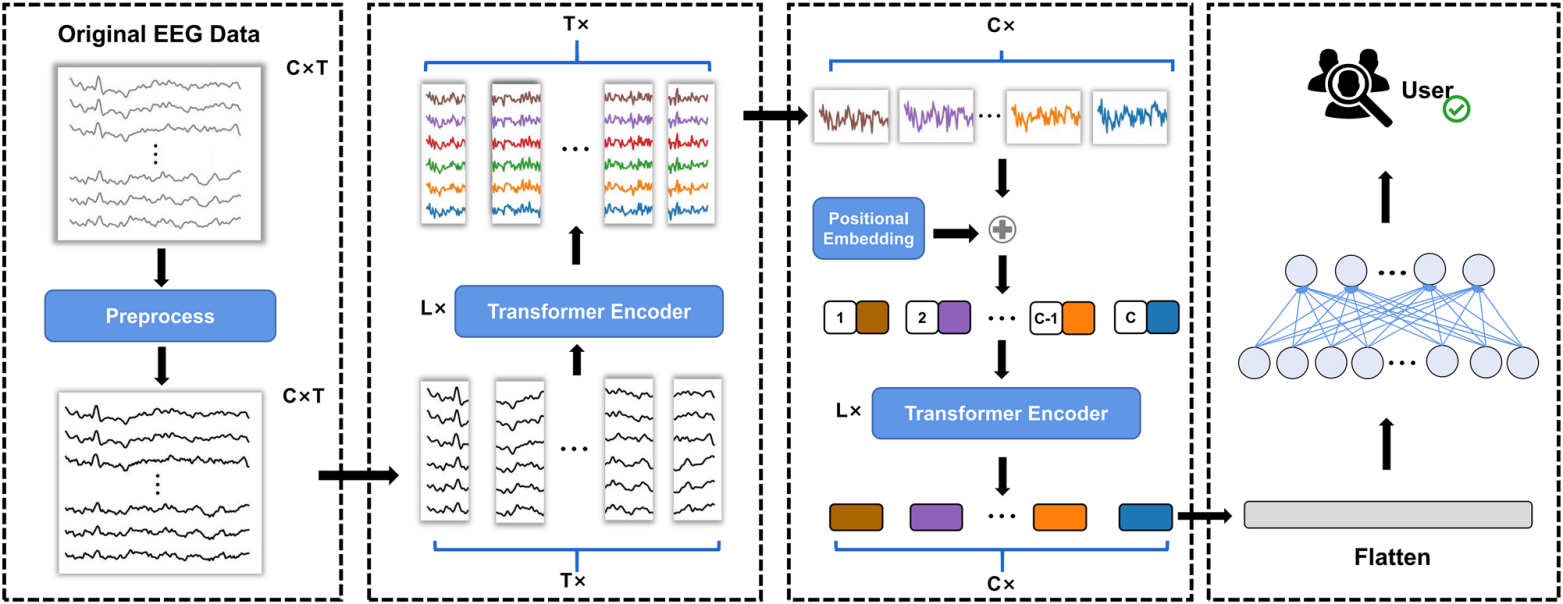
The architecture of the ETST model.

In this paper, we propose an EEG person identification model based on the attention mechanism^21^, and the overall framework diagram is shown in Fig. 1. Unlike other models, our approach does not require additional extraction of artificial features of EEG signals, and only raw EEG signals are used for the identification task. Considering that EEG signal is both continuous in time and functionally connected among channels, we design the model to capture both temporal and spatial features. The model consists of two main parts, containing a temporal transformer encoder (TTE) and a spatial transformer encoder (STE). In the TTE part, we use the attention mechanism in time domain to calculate the correlation among sampling points in samples, which is used to extract the time-domain features of the EEG. Since there is individual specificity in the coupling relationship of channels between individuals, we design the STE part to calculate the spatial domain attention for channels to capture the coupling relationship among different channel signals, which enables the model to identify different individuals more stably based on the specific coupling relationship. Finally, a simple fully connected layer is applied to aggregate global information and perform classification. In the following, we will explain the preprocessing of raw EEG and components of the ETST model in detail.

### Preprocessing

Before feeding data into ETST, we first processed the raw EEG. The original EEG signal is filtered using a [0.5 42] Hz bandpass filter to remove low and high-frequency noises. We remove the ocular and muscular artifacts using independent component analysis (ICA). The size of each sample is T $\times$ C, where T is the number of sampling points and C is the number of EEG channels. For each sample, the following z-score standardization will be employed over time for each channel:

1 $$\begin{aligned} {\hat{x}}_{t,c}=\frac{x_{t,c}-{\overline{x}}_{c}}{\sigma _{c}} \end{aligned}$$

where *t* , *c* in $x_{t,c}$ denotes the sampling point and the channel of the sample, ${\overline{x}}_{c}$ denotes the mean of the sample on channel *c* and $\sigma _{c}$ denotes the standard deviation of the sample on channel *c*. After standardization, the mean of the data on each channel of the sample is 0 and the standard deviation is 1.

### Temporal transformer encoder

**Figure 2:**
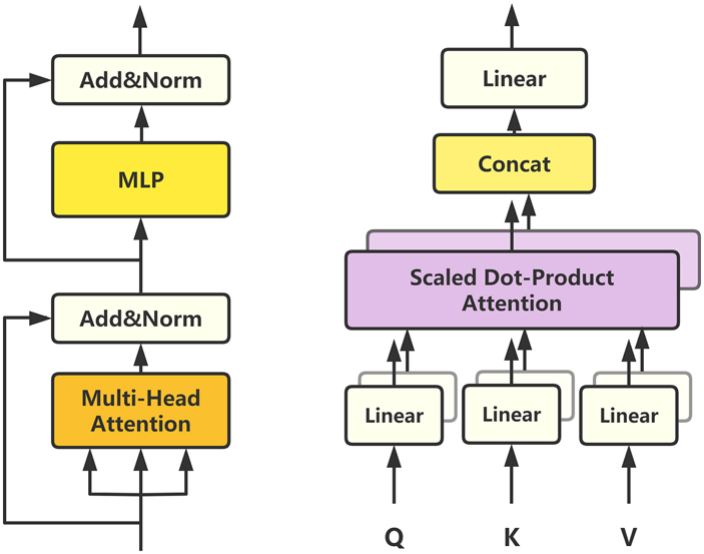
(left) The architecture of a transformer encoder. (right) Multi-head attention.

We use temporal correlation, or correlation between two time points, to capture the time-domain information of EEG signals. Inspired by the attention mechanism^21^, we use multiple transformer blocks to encode the temporal information of the EEG. Instead of convolution focusing on local information, TTE takes into account the long-distance dependence in time. We directly feed EEG data pre-processed into the transformer, instead of employing complicated transformations such as convolutions^28,29^ or trainable linear projections^24^. For a given input $X=[x^{1},x^{2},\ldots ,x^{T}]\in {\mathbb {R}}^{T\times C}$, we compute self-attention in the transformer block to estimate temporal correlations, and then we weight the sum to obtain the new representation. Self-attention is computed as follows:

2 $$\begin{aligned} Attention(Q,K,V)=Softmax\left( \frac{QK^{T}}{\sqrt{d_{k}}}\right) V \end{aligned}$$

where *Q*, *K*, and *V* are all matrices obtained by linear projections of the input and $d_{k}$ is a scalar factor. To jointly attend to information from different representation subspaces at different positions, we adopt the multi-head attention mechanism^21^ on the input. Each transformer encoder contains two parts: multi-head attention (MHA) and multi-layer perceptron (MLP). Each part employs residual connection^30^ and layer normalization (LN)^31^ to improve the speed of training and robustness of the model. Figure 2 illustrates the above calculation process. The TTE part can be expressed by :

3 $$\begin{aligned}&h^{t}_{l}=LN\left( MHA\left( z^{t}_{l-1}\right) +z^{t}_{l-1}\right) \quad l=1,2,\ldots ,L \end{aligned}$$

4 $$\begin{aligned}&z^{t}_{l}=LN\left( MLP\left( h^{t}_{l}\right) +h^{t}_{l}\right) \quad l=1,2,\ldots ,L \end{aligned}$$

### Spatial transformer encoder

The channels in the EEG signal represent the locations of the electrodes on the scalp, and the functional connectivity between different brain regions can be calculated by considering the dependencies among different channels. Similar to TTE, in STE we also used the attention mechanism to model the spatial information among different channels. In order to preserve the spatial location information, we added the position encoding of the spatial domain to the input and then fed the result to STE:

5 $$\begin{aligned} z^{s}_{0}=tran\left( z^{t}_{L}\right) +E_{pos} \end{aligned}$$

where *tran*() represents the transpose operation and the $E_{pos}\in {\mathbb {R}}^{C\times T}$ represents the position encoding. In this paper, we use the position encoding in the form of a trigonometric function at a fixed position. $z^{s}_{0}$ denotes the representation with the addition of spatial position information. In the STE, we use a similar structure to that in the TTE to learn the spatial information on the different channels of the EEG. The process equation is expressed as:

6 $$\begin{aligned}&h^{s}_{l}=LN\left( MHA\left( z^{s}_{l-1}\right) +z^{s}_{l-1}\right) \quad l=1,2,\ldots ,L \end{aligned}$$

7 $$\begin{aligned}&z^{s}_{l}=LN\left( MLP\left( h^{s}_{l}\right) +h^{s}_{l}\right) \quad l=1,2,\ldots ,L \end{aligned}$$

### Classification layer

The output of the transformer encoder layers, TTE and STE, yield a better representation containing both time-domain and space-domain features. ETST learns the time-domain information of the EEG data on the different sampling points in TTE. In the subsequent STE, ETST learns the spatial information among channels. Then, to fuse the global information in the representation for classification, a simple fully-connected layer with only one layer is used to obtain the final classification output which is optimized using the cross-entropy loss function.

8 $$\begin{aligned} L=-\frac{1}{N}\sum ^{N}_{n=1}\sum ^{C}_{c=1}y^{c}_{n}log\left( {\hat{y}}^{c}_{n}\right) \end{aligned}$$

where *N* denotes the number of batch sizes and *C* denotes the number of categories. $y^{c}_{n}$ is the true one hot label, ${\hat{y}}^{c}_{n}$ is the predicted probability of the corresponding category.

### Ethical approval

This paper does not contain any studies with human or animals participants performed by any of the authors.

## Experiments

### Dataset

We validate our method on an EEG dataset provided by PhysioNet^32^. This dataset was recorded using the BCI2000 system^33^ and consists of over 1500 1- and 2-min EEG recordings, obtained from 109 subjects. The sampling frequency was 160 Hz. These EEG data were recorded with 64 electrodes, which conformed to the 10–10 system. Subjects were asked to do motor/imagery tasks while the EEG signal was recorded by the system. Each subject completed 14 experimental runs including 2 1-min baseline runs and 12 2-min task runs. In the baseline runs, the EEG signals were recorded while the subjects kept their eyes open (EO) and eyes closed (EC), respectively. In the task runs, subjects were asked to complete four motor/imagery tasks, including actually completing the corresponding physical action (PHY) or imagine completing the corresponding action (IMA) when the target appeared on the computer, and rest when the target disappeared. Task 1 is to open and clench the corresponding fist when a target is on the left or right side of the computer screen. Task 2 is to imagine opening and clenching the corresponding fist when a target is on the left or right side of the computer screen. Task 3 is to open and clench both fists when a target appears on the top or bottom of the computer. Task 4 is to imagine opening and clenching both fists when a target appears at the top or bottom of the computer. Each task is repeated for three times, totaling twelve task runs. In our experiments, we use all the subjects in the dataset. A 1-s window with 50% overlap of each channel is used to generate samples. Therefore, the shape of a sample is 160 $\times$ 64.

### Experiment design

To make EEG person identification technology realistic and feasible, the stability and robustness of the system must be able to be guaranteed. This also means that the model needs to be able to consistently and accurately identify subjects by their EEG signals, even if the subjects are in different states, such as happy or calm, or even thinking about something. We conducted several experiments to verify the effectiveness and practicability of ETST on EEG biometrics. The EEG signal in the Physionet Dataset contains four states, EO, EC, PHY, and IMA. We designed various experiments based on these four different states to test the performance of ETST in diverse scenarios. The experiments we conducted are described below.

1. We compared our model with state-of-the-art EEG identification methods and also with traditional neural network methods such as CNN, MLP, and traditional machine learning methods such as SVM. In the comparison experiments with other methods, we set up three sub-experiments. The first one is training and testing in a single human state, and we conducted training and testing in four states, EC, EO, IMA, and PHY, which corresponds to the case of EEG person identification in a fixed state. The second one is to train in one state and test in another state, we will train under EC and EO data and test under IMA and PHY. This type of task is the most challenging, and it tests whether the model obtained by training under one EEG paradigm can be generalized to other EEG paradigms. The third one is a mixture of EC, EO, IMA, and PHY datasets for training and testing. For within-state and diverse-states experiments, we randomly divide the dataset into 4:1 as training set and test set respectively.
2. We performed ablation experiments to explore the effect of each part of the model on the results. Position encoding is an important component of the model. The EEG signal contains position information in both the time and space domains. Transformer ensures that the model retains the location information by adding position encoding to the input species. We investigate the effect of adding time-domain position encoding and space-domain position encoding on person identification separately. In addition to comparing spatial and temporal position encodings, we also conducted ablation experiments on the encoder part of ETST. We investigated the performance of ETST when removing TTE and STE respectively, to explore the role of each encoder part.
3. In EEG identification methods, there has not been a consensus on the best segmentation length of samples. For example, the segmentation length used by Wang et al. is 1s^34^, while the segmentation length used by Thiago Schons et al. is 12s^35^, and there may be a large gap between the sample segmentation lengths of different methods. Therefore, we divided the dataset with different split lengths in our experiments for exploring the performance of ETST with different sample split lengths.

In addition to different segmentation lengths, the sample overlap rate also directly affects the size of the resulting sample size and the degree of information overlap among different samples. The loss function of Transformer is smoother than that of CNN^36^, which potentially makes Transformer more difficult to converge with smaller sample sizes, resulting in worse performance. Therefore, we design experiments with different sample overlap lengths and obtained training datasets with different sample sizes to explore the effect of sample size on our model.

### Experiment detail

All experiments in this paper are performed on NVIDIA TITAN Xp GPU. The number of TTE layers, the number of heads of TTE layers, the number of STE layers, and the number of heads of STE layers in the model are set to 2, 8, 2, and 8, respectively. We use the AdamW^37^ optimizer with learning rate, weight decay, and batch size of 4e−5, 1e−6, and 256, respectively, to optimize the network.

## Results and discussion

### Evaluation and comparison with baseline

Currently, EEG-based person identification algorithms are broadly classified into two categories. One is the traditional machine learning algorithms, which generally require manual feature extraction including power spectral density (PSD), auto-regressive coefficient (AR), and fuzzy entropy (FuzzEn). Another category is deep learning algorithms, such as CNN-based or RNN-based neural network models. In addition, since the concept of graph fits well with the functional connectivity in neuroscience, where graph features are used to represent the relationships among brain regions, graph convolutional neural networks (GCNN) are also gaining popularity in the field of EEG. Wang et al. computed Phase Locking Value (PLV) and Pearson’s correlation (COR) as the edge feature between nodes to construct graphs and achieved state-of-the-art results^34^. We compared our method with other advanced methods^15^. Also, we explored the effect of the recent transformer-based models, which combine CNN and attention^38,39^. Therefore, we used the aforementioned methods as the baseline, and compared against the results of our model.

In the first experiment, we investigated the performance of ETST in the same single state. We trained and tested ETST on a single-state dataset to evaluate the mentioned performance.The results are shown in Table 1. The experimental results show that our proposed method outperforms all methods when the data are in the same state , except for one result which is slightly lower than that of GCNN, only 0.2% lower.

**Table 1:** Results of models training and testing within each human state.

Method	EO	EC	PHY	IMA
FuzzEn + SVM^34^	84.14 ± 0.83	83.73 ± 0.71	77.93 ± 0.59	80.84 ± 0.18
Raw + CNN^34^	96.89 ± 0.77	67.43 ± 47.36	97.96 ± 1.55	97.42 ± 0.83
Graph + Mahalanobis distance^15^	99.07 ± 0.19	97.56 ± 0.24	99.74 ± 0.13	99.61 ± 0.11
PLV + GCNN^34^	99.97 ± 0.03	99.88 ± 0.03	**99.99 ± 0.02**	**100.00 ± 0.00**
Lite transformer^38^	83.77 ± 17.39	85.57 ± 22.24	99.76 ± 0.01	99.65 ± 0.08
EA-transformer^39^	98.45 ± 1.17	98.19 ± 3.14	99.93 ± 0.00	99.90 ± 0.01
**Ours**	**100.00 ± 0.00**	**99.96 ± 0.06**	99.97 ± 0.01	**100.00 ± 0.00**

Results are accuracy in testing stage (average  ±  standard deviation)%.

Significant values are in [bold].

The EEG signals can vary drastically under different states, for example, delta waves are associated with increased attention^40^, alpha waves are related with various cognitive features such as task performance^41^, while beta waves are linked to movement or motor imagery^42^. But for EEG biometrics to be practical in real life, the algorithm needs to be robust to state changes. In other words, the model should be able to recognize the identity of the user in different states. Therefore, in the second experiment, we evaluate the generalization ability of our proposed method in different states by training and testing ETST on different datasets. EO and EC data were used as training sets and tested on PHY and IMA data, respectively. Table 2 shows the results of this experiment, which is the training set and test sets are across different states. The results show that ETST has a significant improvement compared to other methods in the condition of different states. Compared with GCNN, the improvements are 10.3% in PHY and 10.27% in IMA. When the states in the training and test sets were different, all methods suffered from performance degradation to a varying degree, with GCNN decreasing by about 13%, SVM by about 40%, and the accuracy of the remaining methods dropping to less than 30%. This indicates that the other models are limited to extracting features from the same states and have weak generalization ability for different states. In contrast, the ETST model only decreases by about 3%, which indicates that the ETST is able to extract features that are valid across diverse states.

**Table 2:** Results of models training on resting states and testing on diverse states.

Method	PHY	IMA
FuzzEn + SVM^34^	16.16 ± 0.01	15.61 ± 0.00
Raw + CNN^34^	49.26 ± 3.85	52.51 ± 2.26
Graph + Mahalanobis distance^15^	69.98 ± 0.38	69.47 ± 0.64
PLV + GCNN^34^	85.40 ± 1.62	87.03 ± 2.53
Lite transformer^38^	87.37 ± 1.10	89.03 ± 0.73
EA-transformer^39^	89.47 ± 0.34	90.66 ± 0.39
**Ours**	**97.29 ± 0.03**	**97.45 ± 0.13**

Results are accuracy in testing stage (average ± standard deviation)%.

Significant values are in [bold].

To enhance the model’s robustness to various mental states, in addition to the strong generalization ability of the model itself, another approach is to include multiple states in the training set and make the model learn to extract features common to all states. Hence, in the third experiment, we included all states in both the training and test sets, including EO, EC, PHY, and IMA. ETST achieves close to the best results, as shown in Table 3. Compared to results from the previous experiment, the results of this experiment show less decrease in accuracy, and only SVM has a considerable decrease, down to 73%. It shows that different algorithms can achieve good results in case the training and test sets contain all states data. However, this enhancement method is not applicable to realistic scenarios. Due to the complexity and variability of human states, it is impossible to contain data of all states in the training set. Therefore, the key to solving the EEG-based person identification problem is to improve the generalization ability of the model among different states. And our proposed ETST possesses a strong generalization ability.

**Table 3:** Results of models training on diverse states and testing on diverse states.

Method	Results
FuzzEn + SVM^34^	73.45 ± 0.10
Raw + CNN^34^	99.85 ± 0.06
Graph + Mahalanobis distance^15^	96.22 ± 0.23
**PLV+GCNN**^34^	**99.98 ± 0.02**
Lite transformer^38^	98.16 ± 0.65
EA-transformer^39^	99.90 ± 0.01
Ours	99.90 ± 0.03

Results are accuracy in testing stage (average  ±  standard deviation)%.

Significant values are in [bold].

### Ablation experiment

In Transformer, self-attention calculates attention weights for all inputs simultaneously and sums the weights to obtain the output. In this process, self-attention considers the global information and discards the location information of the input data. For EEG data, the signal contains location information in both the time and space domains, representing different temporal sampling points and various brain regions, respectively. To investigate the effect of location information in EEG on person identification, we tried retaining the location information of EEG by adding position encoding to the input of TTE and STE layers, respectively. We compare the effect of adding positional encoding to ETST in the time and space domains under the cross-state dataset, and the results are shown in Table 4. It shows that adding only the spatial position encoding produced a better result than that of the temporal position encoding. This model design also yielded the best performance of our model (97% in IMA, 97% in PHY). Adding both temporal and spatial position encoding generated the next best result (96% in IMA, 95% in PHY). We found that the model performance can be improved by adding the spatial information, while diminished by adding temporal information. In addition, by observing the training process of the model, we discovered that adding the location information in the time domain also affects the training efficiency to a certain extent, making the model more likely to converge to worse minima, which leads to bad results. We believe that absolute position encoding in the time domain breaks the translation invariance of EEG signals, thus making it more difficult for the model to extract time-domain features. The absolute spatial position encoding retains the position information of different channels. Unlike the same sampling point that may appear in different locations in adjacent samples, the channel positions in samples are fixed. Thus, the inclusion of absolute position encoding in the space domain could instead improve the model’s ability for spatial feature extraction.

**Table 4:** Results of the ETST model with different position encoding.

Models	PHY	IMA
Non PE	95.84 ± 0.11	96.07 ± 0.03
With temporal PE	79.98 ± 13.03	80.89 ± 12.76
With spatial PE	**97.29 ± 0.03**	**97.45 ± 0.13**
With temporal + spatial PE	90.56 ± 1.94	91.20 ± 1.92

Significant values are in [bold].

The ETST model contains two parts, the TTE layer, and the STE layer, for extracting time-domain and space-domain features, respectively. To illustrate the importance of the two distinct features on the experimental results, we conducted ablation experiments under cross-state for the model to reflect the necessity of each part of our model. As can be seen in Table 5, we compared the results under the TTE, STE, and TTE + STE models. The results indicate that using only the TTE layer or only the STE layer both make the accuracy significantly lower. Moreover, the results show that the TTE layer has a slightly higher classification accuracy than STE (75.19% in IMA and 72.98% in PHY vs. 70.22% in IMA and 68.98% in PHY). Therefore, it can be shown that time domain information is more important than space domain information for person identification. In order to acquire EEG temporal and spatial information simultaneously, our model consists of TTE and STE layers, which can considerably improve the performance of the model and thus achieve the state-of-the-art effect.

**Table 5:** Ablation study on the ETST model (without position encoding).

Models	PHY	IMA
With TTE	72.98 ± 0.39	75.19 ± 0.09
With STE	68.98 ± 0.34	70.22 ± 0.47
With TTE + STE	**95.84 ± 0.11**	**96.07 ± 0.03**

Significant values are in [bold].

### Effect of sample length and sample size

**Figure 3:**
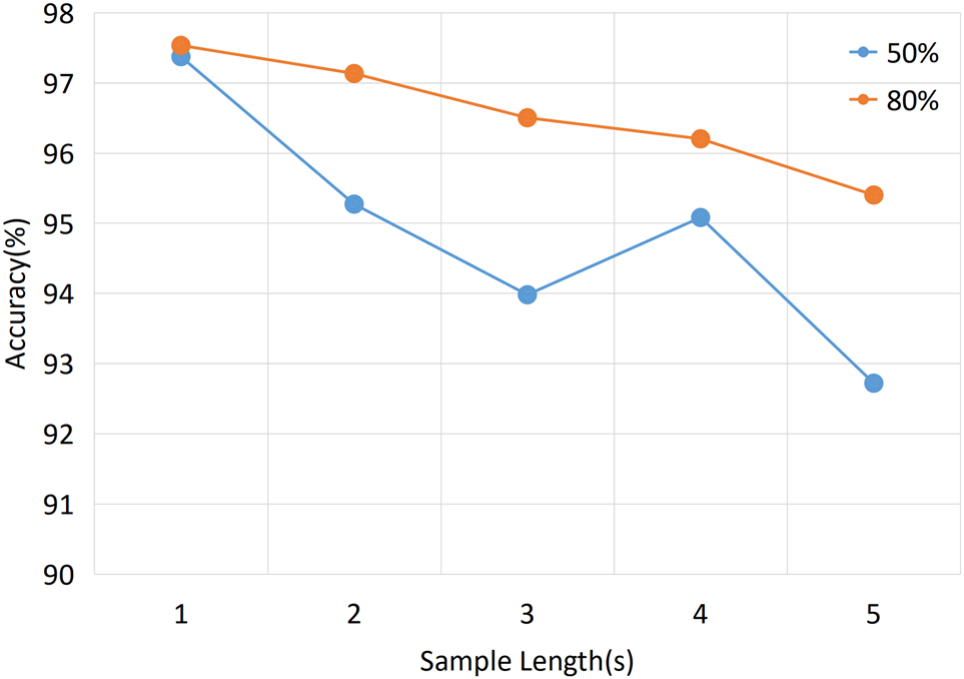
Results of the ETST model in different segment length and overlap.

The sample segmentation length varies in previous methods. As a result, some methods may only work with shorter sample segmentation lengths, while others do the opposite. The same method with samples of different split lengths may yield widely varying results. To illustrate the generalizability in sample length of our method, we compared the classification accuracy of the model under different segmentation length samples. It is worth noting that using a longer sample length would result in a smaller sample size. For example, the sample size of 5-s segmentation length is only about one-fifth of that of 1-s. From Fig. 3, the 1-s length sample achieves the best results with the same overlap rate. Also, we can see that the longer the sample length, the lower the classification accuracy. Namuk Park et al.^36^ mentioned that for Transformer, the size of the dataset directly affects the final training results due to its smoother loss function, i.e., transformer performs worse with fewer samples.

We attempt to increase the number of samples by increasing the overlap rate of the sliding window. Data augmentation of the samples is performed using an overlap rate of 80% and the results are compared for different training set sizes. As seen in Fig. 3, when we changed the overlap ratio to 80%, and thus enlarged the sample size of the dataset by two times, the model accuracy increased. The 5-s accuracy rises to 95.44%, slightly lower by about 2% compared to the 1-s accuracy. This suggests that insufficient sample size of the data worsens the performance of the transformer-based model. In general, regardless of the sample length, our model achieves state-of-the-art results.

## Conclusion

In this paper, we propose ETST, a deep learning model based on the attention mechanism. We used a multi-headed attention mechanism to extract the temporal and spatial features of EEG signals. The temporal transformer encoder in the model is able to extract long-range distinguishable representations, and the spatial transformer encoder is capable to acquire spatial dependencies among channels, which characterizes the functional connectivity among brain regions. In this way, through several rounds of attention weighting, the model is able to focus on the features that are most relevant to the true classification labels. The experimental results indicate that our method achieves state-of-the-art accuracy on person identification, which also validates the feasibility of EEG on biometrics. The model is also robust to different states. The results of the ablation experiments show that the temporal features have a relatively significant effect on the outcome of the EEG biometrics. It also demonstrates that absolute position encoding in space enhances the model. This indicates that specific channels and the correlation among channels can both make an impact on person identification. The experiments demonstrate that longer EEG data lead to a slight decrease in the performance of the attention mechanism. Besides, the application of Transformer in EEG requires sufficient data to ensure its performance. Therefore, it is necessary to investigate the data argument method for EEG data in future studies. In addition, the choice of hyper-parameters for our model is not yet optimal due to the limitation of time, which leads to the suboptimal model performance.

The stability and consistency problems are two key issues in implementing EEG biometrics into practical applications, and there is a need to ensure that the model can re-identify users correctly regardless of conditions and times. This requires the model to be able to extract time-invariant and state-invariant features. In future work, we will explore new approaches to conduct more effective feature extraction for EEG signals. Potential methods include filtering the alpha band features of EEG signals, which has a strong inter-individual variance in the resting state; and selecting the channels with strong correlation to person identification while removing the effect of redundant channels. At the same time, experiments on EEG-based person identification on different days are yet to be conducted.

## Data Availability

The dataset used for this study is publicly available and accessible online at PhysioNet Database [https://physionet.org/content/eegmmidb/1.0.0/]^32^.
